# Obstetrics and Diseases Women and Children

**Published:** 1877-07

**Authors:** Edw. Warren Sawyer


### SECTION II. OBSTETRICS AND DISEASES OF WOMEN AND CHILDREN.

Reported by Edw. Warren Sawyer, M. D.

### First Day, June 5.

Byrd, Ills.—Presented a paper, read by Battey, on some of the diseases requiring dilatation of the female urethra. A number of cases were cited. The first was that of a vascular growth entirely surrounding the meatus of the urethra. It had been treated as a stricture; with the écraseur and scissors a small vascular growth was removed without difficulty. Subsequently dilatation of the urethral canal entirely cured the urethrismus. The case was illustrated by drawings of the parts with the growth in situ and after removal.

Other cases were cited to illustrate the method and ease of dilating the female urethra; for vesical tenesmus, etc.

In the discussion, Marcy, Mass., said that in a case of his, he had removed a growth as large as a cherry; the removal was not dangerous. Three years had now elapsed without a recurrence.

Smith, Ia.—Had removed the growth in two cases with the scissors without difficulty or danger.

Jenks, Mich.—Rose to add that in all instances these growths should be removed, and that no complicated instruments were needed to dilate the female urethra. He used a series of successive sizes of conical rectal bougies until the finger could be easily passed.

Jackson, Chicago.—Thought it often difficult to find these flat and but slightly elevated growths within the urethra. To facilitate diagnosis and removal, he used a conical glass speculum with a fenestrum, into which the growth dropped. The base of the growth he usually touched with fuming nitric acid.

Marcy, Mass.—Said that he had always used the method and speculum spoken of by the last gentleman, thinking that was the usual procedure.

Kimball, Mass.—Presented a paper, read by Dr. Martin, on extirpation of the uterus. After giving an account of the early history and results of the operation, and considering in detail the observations of Péan and Caternault, the writer proceeded to examine the question, Is the operation ever justifiable? It was a sad comment, that nearly nine in ten cases resulted in death. In Scotland all cases were fatal except the three successes of Keith. Death is caused by shock, hemorrhage, inflammation and septicæmia.

A case was detailed, which the reader said might be called a specimen case. A woman had presented herself to him with a hard, movable tumor, measuring seven or eight inches in its longest diameter, which was easily recognized as an interstitial fibroid. The patient was sent away, and told that nothing could be done for her. Two years afterwards she returned, but so changed that she was not recognized. Nor was the diagnosis of the tumor at that time made out. The patient insisted upon an operation which was undertaken under the conviction that the tumor was as likely to be ovarian as uterine. Upon opening the abdomen, an unusual appearance was presented by the omentum, which had never before been met with by the writer; viz., innumerable cysts, varying in size from a robin's egg to a pea, everywhere studded the omentum. The omental mass was strangulated and cut off. The entire uterine mass was now removed; it weighed thirty-one pounds. The long pedicle was stitched into the lower end of the abdominal incision. Recovery was perfect at the end of the twelfth week.

Without denouncing the operation, it is one which is justifiable only in extremely rare instances. During a period of twenty years, it seemed justifiable in but two or three instances. Undoubtedly it is most often performed on account of an error in diagnosis.

Martin, Mass.—Spoke of the noted New England case in which it was not known, till after the operation, that the uterus had been removed; the operation was recovered from.

Kimball, Mass.—Feared it might look like vanity, but the first operation which he did in 1853 was the first, so far as he knew, which was undertaken deliberately—the diagnosis being established. Altogether, he had operated twelve times with six recoveries. One was an instance of pedunculated fibroid, and in another instance the uterus was amputated through its body. Trenholme had once removed the uterus on account of the rupture of a cyst with symptoms of septicæmia. The woman died.

The writer spoke of one class of cases in which the operation was especially contraindicated: those in which the growth of the tumor was downward, occupying the pelvic excavation.

Reamey, O.—Alluded to a series of five cases belonging to

Dr. Thomas Wood, of Ohio, in which there were three recoveries.

Smith, Iowa.—Had once performed the operation under an error in diagnosis. A trocar was driven into the intumescence which drained away some two ounces of amber colored liquid; it was thought to be from an ovarian cyst, and gastrotomy was performed. The tumor was found to be uterine, and the mass removed; it weighed fifteen pounds. Upon the upper surface of the growth a collapsed cyst was found, into which the trocar entered. The woman died on the sixth day.

Sims, N. Y.—Regarded the paper a valuable contribution to the literature of the subject, and its appearance now as most timely; especially as some are carried away with the idea that the operation should be performed much more often. He believed the operation was sometimes justifiable; but it was not always so in Péan's cases. He had performed the operation three times; in two instances the patients died of shock, and the third instance died from septicæmia. In this case there was sloughing of the broad ligament, and some eighteen ounces of pus were found in the abdominal cavity.

Jennings, Ark.—Inquired of Dr. Kimball what per centage of cases died of septicæmia.

Kimball, Mass.—Peritonitis and septicæmia usually co-exist. One case died from hemorrhage; the rest died from peritonitis and septicæmia. In one case there was a most singular manifestation of septicæmia: the patient had survived three weeks, when a lump appeared in one parotid region which grew so rapidly that the poor woman was asphyxiated.

Marcy, Mass.—How long after the recovery from the operation were the patients alive?

Kimball, Mass.—So far as he knew they were all now alive, except in one instance. In one case he had removed an ovarian tumor eleven years before ablating the uterus. In another subject he removed the ovaries with the uterus.

Atter, Pa.—Was physically unable to to take part in the discussion of this interesting subject. He wished to say that, in his opinion, the operation should never be performed, save when death was inevitable, and always as a *dernier resort*.

Byford, Ills.—Agreed with all who had spoken. He believed the operation is never justifiable except as a last expedient, and before deciding that the operation should be performed in a given case, we should separate the cases in which death is really imminent from those in which it is only apparently so. For instance, it is well known that these uterine tumors are often the seat of inflammation, when it would seem that the patients life was endangered; but inflammation is a process by which nature lessens the tumor and sometimes causes it to disappear, and is often recovered from. The danger of death from exhaustion, and especially from pressure upon the neighboring vital viscera are among the indications which might justify the removal of the uterus.

Grant, Ca.—Had listened with great pleasure to the paper and the discussion. So far as he knew, the opportunities for observation upon this subject had been extremely rare in Canada. He had known of but two cases: one followed by death, and one by recovery.

Mary Thomas, Ind.—Knew of one instance of recovery from removal of uterus, but thought the operation should be resorted to with great hesitation.

White, N. Y.—Rose to thank Dr. Kimball for his very able paper; very few men, however, who had had such success, were as modest. In other words, the success attained by Dr. Kimball hardly warranted the conclusion that the operation is almost never justifiable, which had been expressed by so many present. Why wait till death is inevitable, and the patient moribund? Why not give the patient the advantage of her only hope, at a time when the operation promises most? He believed in going forward. The early ovariotomists were greatly opposed, and the whole world was slow in accepting, what had been demonstrated in America, that ovariotomy was a justifiable operation. The operation under consideration has a great future. He declared himself conservative but not timid. Three cases had occurred in his practice. Some weeks since, he was sent for to do the operation, but the order was countermanded because the woman was supposed to be dying. The woman did not die, and he was again sent for and removed an immense fibro-cystic tumor; the woman died from shock. A year earlier the woman might have been saved by the operation. In another case, the woman died from exhaustion. The third case was operated upon under an error of diagnosis; it proved to be uterine instead of ovarian, and the uterus was removed through the neck. This woman recovered.

Bozeman, N. Y.—Read a paper entitled Kolpokleisis, as a means of treating vesico-vaginal fistula; is the procedure ever necessary? The conclusions of the writer, were, that by a systematic and proper preparatory treatment, vesico-vaginal fistula can always be cured by operation, without ever necessitating the destruction of the vagina. The paper consisted chiefly of a correspondence between Prof. Simon, of Heidelberg, and the reader, upon a series of cases upon which both had operated in concourse, in Heidelberg. The reader exhibited his graduated set of vaginal dilators, the systematic use of which distinguishes the author's operations.

### Second Day, June 6.

Bozeman, N. Y., wished to read some statistics in addition to his paper of yesterday. Of twenty-five cases of vesico-vaginal fistula, collected from European journals, eighteen were condemned to kolpokleisis; two died from the operation. The paper was not discussed.

Marcy, Mass., presented a paper on the congenital absence of the uterus. Several interesting instances were cited. One, aged 29, had never menstruated; had no pubic or axillary hair; externally was well formed; had no sexual instinct. There was a vagina, but every mode of examination failed to elicit the existence of the uterus. She suffered regularly from symptoms of suppressed menstrual flow. The woman had two sisters who had similar evidences of this imperfect development. In another case spoken of in the paper, the woman, aged 32, had no periodic manifestations. In a third instance, there was a slight uterine mass which was threaded, as it were, by the passing sound.

Webber, Ind.—Had recently seen a woman, married for two years, who had never menstruated, and in whom he could find no signs of a uterus.

Staples, Minn.—Had recently dissected a fully grown fœtus, whose external parts appeared those of a female. There was no vagina and no uterus; ovaries present. No anus; the intestine ended in the bladder.

Warner, Mass.—Had met with an instance in which neither vagina or uterus existed. The woman was otherwise perfectly formed.

Sims, N. Y.—Had met with five cases; but, unlike Dr. Marcy's case, the vagina in every instance was wanting. In all instances the subjects seemed perfectly formed, and one, particularly, was a beautiful woman. But, in all instances, the uterus and vagina were absent. In one instance the subject was madly in love, but marriage was of course interdicted. In regard to the sexual instinct, the speaker said he had now been in practice forty-two years, and had never been guilty of asking a woman if she experienced the sexual desire.

Dean, N. Y.—Spoke of the liability of error in deciding that there was no uterus. A case was detailed; that of a girl, aged 15, who had not menstruated. Vagina imperfectly formed, but not positively wanting. The entire staff of the Rochester hospital agreed that the uterus was absent. Subsequently, an ill defined tumor was felt above the symphysis pubis. An incision was made upward in the direction of the vagina which revealed an os and uterus, from which drained away a quantity of retained menstrual matter. The girl has since menstruated.

Bozeman, N. Y.—Mentioned a case, otherwise perfectly formed, but in which the uterus and sexual instinct were wanting.

Seymour, Troy, N. Y.—Mentioned a case in which the uterus was not altogether wanting, but in which the growth of the organ had been early arrested, perhaps by a severe attack of scarlatina which she suffered in her childhood. She had never menstruated, and the sexual instinct, and female modesty and shame, were entirely wanting. It occurred to him that the uterus might be made to grow; and, with this end in view, stimulating injections were used; soon the uterus began to grow, and the woman afterward began to menstruate.

Subsequently her womanly instincts began to predominate, and rather to his detriment, for she blackened his reputation in a large section of his city by detailing everything that had been said and done to her during her treatment. The speaker protested against its being declared indelicate to ask a woman concerning her sexual feelings, or concerning anything that can throw light upon the case under consideration. "In the name of God is it more impertinent to inquire about the sexual feelings than to know about the perfectly formed external parts?"

Parvin, Ind.—Had met with one case in which there was no evidence of a uterus. He spoke particularly of a class of cases in which the uterus is not absent but undeveloped; the function of menstruation is sometimes absent and sometimes vicarious. The uterine mass is often no larger than the end of the little finger. Passing the sound into its minute opening is quite like threading the mass, as Dr. Marcy very aptly said. Such cases are important, because amenable to treatment, and the development of the uterus can sometimes be artificially completed. The speaker confirmed Dr. Sims in his views of the indelicacy of asking a woman concerning her sexual feelings. He knew not how to ask such questions. He was ignorant of the euphony with which to clothe the thought. "I doubt if ancient Troy would have fallen had it contained one with the heroism of this modern Trojan."

Battey, Ga.—Had seen four cases in which the uterus was apparently absent. In one the vagina was about an inch and a half long; no uterus was felt; the woman was barren, and had never menstruated.

White, N. Y.—Had seen ten or more such cases. One woman had previously consulted a practitioner because coitus was imperfectly executed. She was assured that the deformity could be remedied, and submitted to an operation. A hole was made, which satisfied her husband, but she was not able afterwards to hold her water, which was constantly running away. An examination showed absence of vagina and uterus, and a large hole in her bladder. This vesical fistula the speaker closed by operation. In this case, when the finger was passed into the bladder, the ovaries could be easily felt upon either side. In another case the creature had been brought to him to be fitted with a truss. He found what appeared to be the labia of the vulva, and one labium contained a testicle, upon which the truss was to be fitted. In still another instance, he found the labia containing testicles, the subject being brought to him to decide whether the creature should be christened George or Georgiana.

Byford, Ill.—The experience of the gentlemen who had spoken surprised him; he was not before aware that there were so many women in the world without a uterus. Two cases occurring under his observation were spoken of; in each there was an undeveloped uterus; in one galvanism was tried without result; the subject was more than thirty years old. In another case the uterus was at first about an inch and a half in length, but it subsequently suppurated and dwindled in size. In all, he had seen some eight doubtful cases.

Crawford, Ill.—In this connection mentioned the case of a child, now eighteen months old, who had menstruated every twenty-eight days for seven successive periods. The period is four days in duration. The amount of blood lost is nearly that lost by the adult. In all other respects she is like other infants.

Reamy, O.—Mentioned a case in which menstruation had never occurred till the age of 21. By operation, a vagina was made and the presence of the uterus made out. He protested against the declaration going out from this body that it was indelicate or impertinent to ask a woman concerning her sexual tastes. It is our duty to inquire concerning everything which can throw light upon the case under consideration.

Cutler, Mass.—Was requested to exhibit the electrolytic apparatus devised by him for the electrolysis of uterine tumors. The success of the operation has been greater since he devised the new grooved electrode. These pointed electrodes are driven into the most accessible part of the mass; sometimes directly through the abdominal walls, at other times through the vagina or rectum. The electrodes must always be separated from each other by at least a half inch of tissue; more than this is better. The operation should always be performed under ether. The duration of the sitting varies from five to fifteen minutes.

Hildreth, W. Va.—Exhibited a new vaginal speculum which he had improvised. Its shape is like the common glass speculum, but its walls are made of wires, which run lengthwise, and are separated from each other by half inch spaces. The advantage possessed by it is that the vaginal wall can be seen at any point.

THIRD DAY, JUNE 7.

Smith, Ia.—Read a paper on how to decide the best position in every labor. The reader had been early convinced, by a case of extreme lateral obliquity of the womb, that the position of the woman had much to do with the progress of labor. In every case, we should carefully find out the relations of the axis of the gravid uterus to the entrance of the parturient canal, and place the woman in that position which is most favorable to the entrance of the fœtus into the pelvis.

Parker, Mass.—Reported a case of a large growth connected with the clitoris, which was removed. The subject was a prostitute, aged thirty-five years. She had been insane more than three years. It was known that, at least three years ago, the tumor of the clitoris was as large as an orange. It was now much larger. Under ether the growth was removed without difficulty, with the écraseur. The mass weighed four and a half pounds. Its gross appearances were those of a syphilitic condyloma.

Immediately upon recovering from the effects of ether, she was apparently perfectly sane, and remained so. She exclaimed to the operator: "God bless you, why have you not done this before?"

She also said that she had another trouble; viz., her stomach. A fullness over the region had been often noticed. During her stay in the alms-house, she had been observed to vomit frequently. She had a morbid predilection for cabbage, which she would eat in great quantity and immediately reject.

The patient died three days after the removal of the tumor, though from no effects of the operation. An autopsy revealed the stomach nearly full of hay, rolled in a large circular mass, and one large ball had been recently driven through the pyloric orifice of the stomach, which had probably caused death.

Photographs were shown of the tumor in situ, and of the stomach on section containing the hay.

White, N. Y.—Completed reading his annual report on obstetrics and gynecology, which had been presented at the morning session of the Association.

The reader dwelt particularly upon the treatment of inversion of the uterus, by gradual reposition. By means of the appliance used by him, the reader said that all inverted uteri could be replaced; no matter the length of duration of the inversion—thirty years or six months. The paper concluded with a plea for bed-side instruction in obstetrics; which system the reader instituted in this country more than thirty years ago.

Sims, N. Y.—Contrasted the infrequent use of the forceps in labor years ago, with the humane use of the instrument at this time. Some years ago, Dr. Quackenbush, then quite a young man, startled the N. Y. State Society by saying that he had used the forceps in fifteen hundred labors. This was perhaps the beginning of the frequent use of the instrument, which now prevails in this country, but to no such degree abroad. The speaker alluded to a paper by Dr. Newman, of Denver, in which the use of short forceps was advocated in the last moments of labor. A very short forceps was exhibited, which the speaker thought (erroneously) was devised by Dr. Newman to be used in the last moments of labor.

Quimby, N. J.—Spoke against such frequent use of the forceps; it is meddlesome and dangerous midwifery. The case should be left to nature for at least six or eight hours. The forceps should only be used when nature shows signs of failing.

Fairbanks, Mich.—No ignorant practitioner should be entrusted with the use of the instrument. Thirty years ago a practioner would have been condemned and called a butcher if he had used the forceps.

Jenks, Mich.—Said it was no argument against the use of an instrument, that, in the hands of unqualified men, it could do harm. He should be sorry if the teaching now prevailed of waiting as was taught formerly. When the parts are ready for delivery, there can be no advantage in waiting. Under these circumstances, delay is an indication for the use of the forceps.

Reamy, O.—Said the time had gone by of measuring the powers of nature by the watch. The intelligent practitioner should be ready to interfere at any moment and at any stage of the labor. Damage to the woman and child is caused much more often by too great delay, than by the too early use of the forceps. The speaker said, of the particular forceps now before the section, that it possessed many faults, which he pointed out, and concluded by saying, that it was a beautiful instrument, but at the same time it was a useless thing—a mere toy.

Woodward, Vt.—Also thought the little instrument in question a useless instrument, and spoke of the advantages of the Hodge forceps. He was formerly taught to use the forceps to save the child; but he also used it now to save the woman's bladder, which he did by its early use.

Battey, Ga.—Said that it had been claimed as an advantage, that this forceps could be applied without the knowledge of the woman. He had more than once been guilty of asking the question which had been so severely condemned yesterday, but had never used the forceps clandestinely.

Byford, Ill.—Said that in his practice, when the pains fail and the head does not advance, he interfered without reference to time. He wished to defend the little instrument which had been so severely abused. The criticisms made by Dr. Reamy, might apply to Dr. Newman's forceps, but the instrument now exhibited is not Newman's forceps but Sawyer's forceps, which was a modification of Newman's and a hundred per cent. better than the original. He had seen the instrument in actual use, and its excellence was apparent. He had no hesitation in saying that, for the class of cases which the author had in mind, it was the best short forceps in the world.

Garnish, Ind.—Protested against such teaching going out from this body, advising such frequent use of the forceps. During a practice of twenty-five years, in but ten or twelve cases did he use the forceps. The instrument should be reserved for those cases in which nature has failed.

Sims, N. Y.—Was specially asked to give his views upon the treatment of stenosis uteri. The speaker detailed the history of the surgical treatment of the affection; Simpson was its author, who claimed that no damage resulted from the operation; but in two weeks from its introduction in New York, two cases of dangerous hemorrhage had resulted in his practice. Subsequently, when the speaker visited Edinburgh, he found that accidents did occur, even in the hands of the great master himself.

As first performed, the operation consisted in cutting through the vaginal cervix upon both sides, with the metrotome. Soon after he began to do the operation, a case presented itself to his notice of extreme retro-flexion, that admitted the sound with the greatest difficulty. It occurred to the speaker to amputate the posterior lip of the cervix, which was greatly elongated and with the knife to cut along the posterior aspect of the cervical canal. This was the origin of the operation which is known as Emmet's operation, but which is really Sims' operation, and which he wished known as such. In the operation which he now performs, the knife is used, and the scissors is discarded. If the lips of the cervix are symmetrically developed, the lateral incisions are made with the knife; but if the posterior lip is elongated, the posterior incision is made. The depth of the incision varies with the size of the neck; the substance is cut nearly two-thirds through.

The speaker is particular to guard against all possibility of hemorrhage, by tamponing the cervix with cotton soaked with Liq. Ferri Subsulphas 1 part, water 2 parts. The success of the operation depends upon the subsequent dilatation of the cervix. All operations will be futile, unless a systematic dilatation of the cervix is carried out.

Parvin, Ind.—-Reminded the section that other matters were treated of in Prof. White's report, that merited discussion. In regard to the induction of premature labor in cases of placenta previa, he wished to say that, in his opinion, the procedure should never be resorted to, save when the child is viable and the woman is in great danger from imminent hemorrhage.

Seeley, Ill.—Exhibited a new dilator. It consisted of a silk bag outside of a rubber sack. Any shape can be made which is desired. A tube is carried into the sack to its fundus, for the purpose of holding a metallic sound, by means of which, the dilator is held within the cervical cavity to be dilated. A second tube connects the dilator with the syringe, by means of which the instrument is distended. A disadvantage possessed by the Barnes' dilator is, that it slips out of the canal; in this instrument the sound secures its retention.

Sims, N. Y.—Exhibited Fowler's pessary, which the speaker called almost an universal pessary. In principle, it is like the Hodge, but is much more bulky, being a deep, cup-like shell of vulcanite.

Fitch, Ill.—Pointed out several defects possessed by the instrument; chief of which was its great bulk. Its large surface bearing upon the vagina, would do injury to the part. The opening in the anterior part of the instrument, to facilitate its removal, was a fault; because, through it there was danger of the soft parts prolapsing and becoming strangulated.

